# Supplementary material for: Venomix: a simple bioinformatic pipeline for identifying and characterizing toxin gene candidates from transcriptomic data
Source: PeerJ. 2018 Jul 31;6:e5361. doi: 10.7717/peerj.5361 (PMC6074769; doi:10.7717/peerj.5361)
Supplement: Supplemental Information 3 [file peerj-06-5361-s003.gz › FinalOutput_E-6/Kunitz-type_proteinase_inhibitor_AEPI-IV_2/finaltree.pdf]

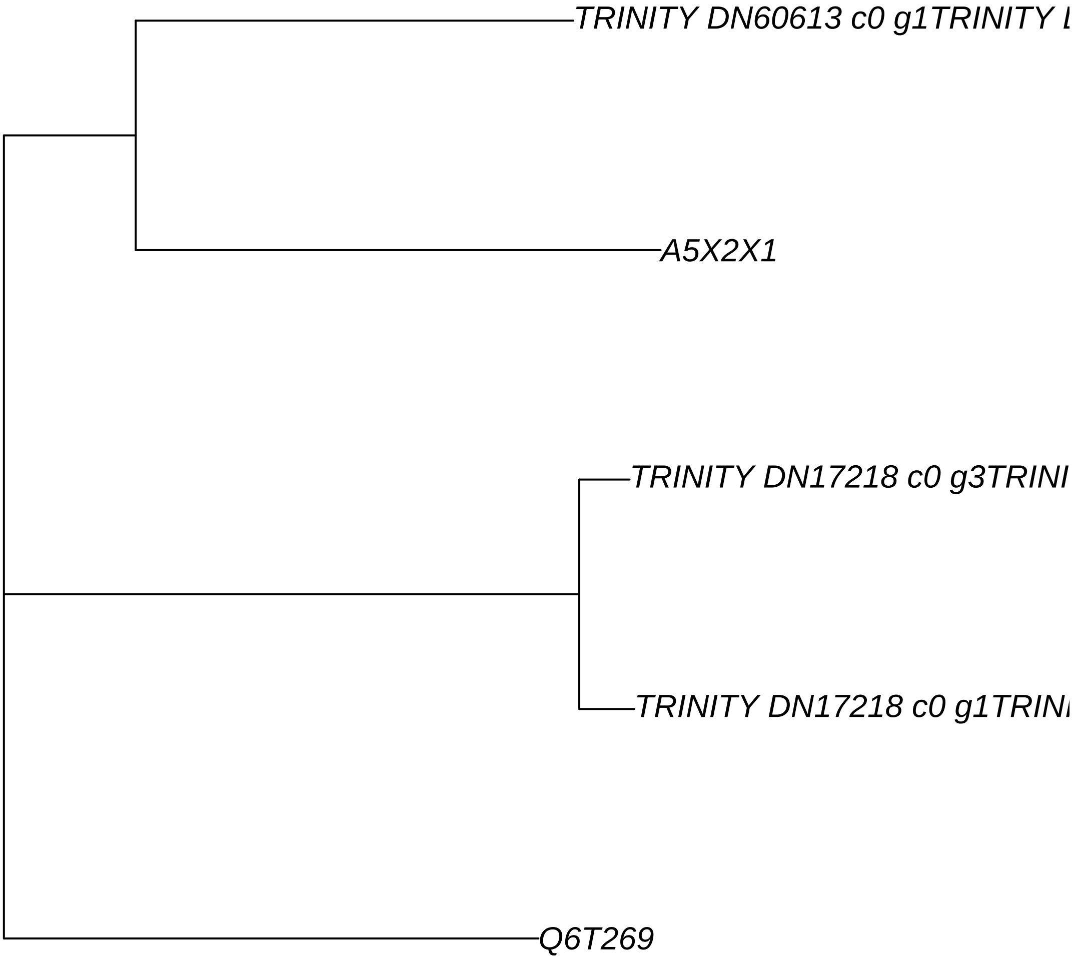
TRINITY DN60613 c0 g1TRINITY D
A5X2X1
TRINITY DN17218 c0 g3TRINI
TRINITY DN17218 c0 g1TRINI
Q6T269
